# A Novel Polymorphism in the Promoter of the *CYP4A11* Gene Is Associated with Susceptibility to Coronary Artery Disease

**DOI:** 10.1155/2018/5812802

**Published:** 2018-02-01

**Authors:** Svetlana Sirotina, Irina Ponomarenko, Alexander Kharchenko, Marina Bykanova, Anna Bocharova, Kseniya Vagaytseva, Vadim Stepanov, Mikhail Churnosov, Maria Solodilova, Alexey Polonikov

**Affiliations:** ^1^Department of Biology, Medical Genetics and Ecology, Kursk State Medical University, Karl Marx Street 3, Kursk 305041, Russia; ^2^Department of Internal Medicine, Kursk State Medical University, 14 Pirogova St., Kursk 305035, Russia; ^3^Laboratory of Genomic Research, Research Institute for Genetic and Molecular Epidemiology, Kursk State Medical University, Yamskaya Street 18, Kursk 305041, Russia; ^4^Evolutionary Genetics Laboratory, Research Institute of Medical Genetics, Tomsk National Research Medical Center, 10 Nabereznaya Ushaiki, Tomsk 634050, Russia; ^5^Department of Medical Biological Disciplines, Belgorod State University, 85 Pobeda St., Belgorod 308015, Russia; ^6^Laboratory of Statistical Genetics and Bioinformatics, Research Institute for Genetic and Molecular Epidemiology, Kursk State Medical University, 18 Yamskaya St., Kursk 305041, Russia

## Abstract

Enzymes CYP4A11 and CYP4F2 are involved in biosynthesis of vasoactive 20-hydroxyeicosatetraenoic acid and may contribute to pathogenesis of coronary artery disease (CAD). We investigated whether polymorphisms of the *CYP4A11* and *CYP4F2* genes are associated with the risk of CAD in Russian population. DNA samples from 1323 unrelated subjects (637 angiographically confirmed CAD patients and 686 age- and sex-matched healthy individuals) were genotyped for polymorphisms rs3890011, rs9332978, and rs9333029 of *CYP4A11* and rs3093098 and rs1558139 of *CYP4F2* by using the Mass-ARRAY 4 system. SNPs rs3890011 and rs9332978 of *CYP4A11* were associated with increased risk of CAD in women: OR = 1.26, 95% CI: 1.02–1.57, *P* = 0.004, and *Q* = 0.01 and OR = 1.45, 95% CI: 1.13–1.87, *P* = 0.004, and *Q* = 0.01, respectively. Haplotype G-C-A of *CYP4A11* was associated with increased risk of CAD (adjusted OR = 1.41, 95% CI: 1.12–1.78, and *P* = 0.0036). Epistatic interactions were found between rs9332978 of *CYP4A11* and rs1558139 of *CYP4F2* (*P*_interaction_ = 0.025). In silico analysis allowed identifying that SNP rs9332978 is located at a binding site for multiple transcription factors; many of them are known to regulate the pathways involved in the pathogenesis of CAD. This is the first study in Europeans that reported association between polymorphism rs9332978 of *CYP4A11* and susceptibility to coronary artery disease.

## 1. Introduction

Coronary artery disease (CAD) is a common cardiovascular disorder (CVD), a major cause of mortality and disability in Russia and worldwide [1, 2]. CAD is a multifactorial polygenic disorder resulting from complex interactions between multiple genetic and environmental factors [3, 4]. Advances in molecular genetic and biochemical techniques have improved our understanding of the metabolic disorders causing CVD and coronary atherosclerosis, and the identification of candidate genes responsible for CAD susceptibility is now an area of intense research interest. Genome-wide association studies (GWAS) have provided powerful tools to dissect genetic determinants of complex multifactorial disorders and to identify new potential genes that may increase the risk of coronary artery disease. Meta-analyses of the largest GWAS conducted on coronary artery disease have identified a number of genes associated with disease susceptibility in different populations and provided insights into the molecular basis of the disease [5, 6].

Genetically determined alterations in the metabolism of arachidonic acid (AA) have been implicated in the pathogenesis of CVD such as hypertension, atherosclerosis, and coronary artery disease [7–10]. Arachidonic acid is metabolized by various enzymes such as cyclooxygenases, lipoxygenases, and cytochrome P450 monooxygenases producing a variety of bioactive substances such as prostanoids, leukotrienes, hydroxyeicosatetraenoic acids (HETEs), and epoxyeicosatrienoic acids (EETs) [11, 12]. EETs are products of cytochrome P450 epoxygenases that realize their cardiovascular effects through activating receptor-mediated signaling pathways and ion channels and possess vasodilatory, angiogenic, and anti-inflammatory properties in the cardiovascular system [7, 8, 13, 14]. 20-HETEs (20-hydroxyeicosatetraenoic acids) are vasoactive eicosanoids which are derived from the *ω*-hydroxylation of AA by members of the CYP4 gene family and known to be involved in the regulation of vascular tone and sodium transport in the kidney [10, 15]. 20-HETEs possess multifaceted effects on cardiovascular functions including those implicated to the pathogenesis of CVD: stimulation of smooth muscle contractility, migration, and proliferation, as well as activation of endothelial cell dysfunction, angiogenesis, and inflammation [9, 15]. Cytochrome P450 4A11 and 4F2 are the major 20-HETE-producing CYP4 isoforms in humans [16, 17] which also participate in the metabolism of several drugs including those used for therapy of CVDs [18].

Various studies have revealed that single nucleotide polymorphisms (SNPs) in *CYP4A11* and *CYP4F2* have an impact on expression or catalytic activity of these enzymes, thereby contributing to the molecular basis of cardiovascular disorders including CAD [17, 19–24]. Many of these studies have discovered an association between some of these SNPs and the susceptibility to hypertension and coronary artery disease, making *CYP4A11* and *CYP4F2* reasonable candidate genes for altering the risk of CVD. However, the results of these studies were variable and sometimes contradictory that may arise from differences in ethnic backgrounds, effects of environmental factors, or inconsistent inclusion criteria. A huge portion of the studies conducted in Asian populations have considered the *CYP4F2* and *CYP4A11* genes as candidates for pharmacogenetic investigations of drugs. A limited number of studies investigated the contribution of these genes to the development of hypertension [17, 25, 26], and no studies investigated the relationship of these genes with CAD susceptibility in European populations. The purpose of this study was to investigate whether common single nucleotide polymorphisms in the *CYP4A11* and *CYP4F2* genes are associated with susceptibility to coronary artery disease in Russian population.

## 2. Methods

### 2.1. Study Subjects

The study was approved by the Ethical Review Committee of Kursk State Medical University, and the participants who were recruited gave written informed consent. A total of 1323 unrelated Russian subjects including 637 patients with coronary artery disease and 686 healthy controls were enrolled from the Cardiology Divisions of Kursk Regional Clinical Hospital and Kursk Emergency Hospital as well as from the Regional Cardiovascular Centre during a period between 2012 and 2015. All recruited patients had clinical signs or a history of CAD (angina or myocardial infarction) and angiographically confirmed coronary artery stenosis of >50%. CAD patients had no clinical signs and/or histories of congenital heart disease, cardiomyopathy, malignancy, connective-tissue disorder, chronic inflammatory disease, and liver or kidney disease. The control group included blood donors, healthy volunteers without any chronic disease, and also hospital-based patients having no clinical evidence for CAD or a history of cerebrovascular/peripheral vessel disease. These subjects were recruited over several periods in the framework of our previous studies [27–29]. Demographic and clinical data of the study participants are shown in Table 1. As can be seen from Table 1, the study groups were matched with respect to both sex and age (*P* > 0.05). A percentage of positive family history of CAD, hypertension, and diabetes was significantly higher in the case group versus that in the healthy controls. Biochemical parameters (blood lipids and fasting glucose) were available from 347 subjects of the control group. Significant differences between the groups were seen regarding the lipid parameters and blood glucose concentration (Table 1).

### 2.2. Selection of Single Nucleotide Polymorphisms

Six common SNPs such as rs3890011, rs1126742, rs9332978, and rs9333029 of the *CYP4A11* gene and rs3093098 and rs1558139 of the *CYP4F2* gene were selected for the study based on their known functional relevance, haplotype tagging properties, and previously reported associations with cardiovascular diseases [21, 22, 24]. The functionality of the selected SNPs and their haplotype properties were assessed in silico by the SNP Function Prediction tool developed by Xu and Taylor [30] and available online at the SNPinfo Web Server (https://snpinfo.niehs.nih.gov/snpinfo/). SNP rs1126742 of *CYP4A11* was excluded from the study because of insufficient genotyping call rate (<70%) for this polymorphism.

### 2.3. Genotyping

Genomic DNA was isolated from 5 ml of peripheral blood samples obtained from all study participants using standard phenol/chloroform procedure. Polymerase chain reaction (PCR) was performed on the CFX96 Touch™ Real-Time PCR Detection System (Bio-Rad Laboratories, USA). SNP genotyping was performed using a MALDI-TOF mass spectrometry iPLEX platform (Agena Bioscience Inc., San Diego, CA, USA) at the Core Facility “Medical Genomics” in the Research Institute of Medical Genetics (Tomsk, Russia). Blind replicates were included for quality control. Genotype data on SNPs rs9332978 and rs9333029 of *CYP4A11* were not available from two CAD patients and two healthy controls, respectively.

### 2.4. Data Analysis

An association analysis between SNPs and disease risk could detect a difference of 2–6% in the genotype distributions between the cases and controls assuming 81–92% statistical power and a 5% type I error (*α* = 0.05) on the basis of the sample sizes of 637 CAD patients and 686 healthy controls. Allele frequencies were estimated by the gene counting method, and the chi-square test was used to assess significant departures from Hardy–Weinberg equilibrium (HWE). Categorical variables were also compared by using the chi-square test. Allele, genotype, and haplotype frequencies in the study groups were evaluated by the SNPassoc package for R [31] and the SNPStats software [32]. The strength of the association of the SNPs with the occurrence of coronary artery disease was measured by multiple logistic regression analysis to calculate odds ratios (OR) with 95% confidence intervals (CI) and adjusted for confounding factors. Epistatic interactions between SNPs (log-likelihood ratio test (LRT)) were analyzed by the SNPassoc package for R [31], assuming codominant, dominant, and recessive models, and adjusted for age, gender, and hypertension. Haplotypes of *CYP4A11* and *CYP4F2* were estimated in the entire groups of CAD patients and controls by the SNPStats software. *P* value ≤ 0.05 was set to be statistically significant. As an adjustment for multiple testing, false discovery rate- (FDR-) based *Q* value was calculated for each SNP using the method proposed by Benjamini and Hochberg [33] and implemented in the FDR calculator available online at http://www.sdmproject.com/utilities/?show=FDR. Significance of the associations was assessed by a 0.20 threshold of *Q* value, as previously suggested [34]. The regulatory potential of the studied SNPs was evaluated by the SNP Function Prediction tool [30] using the TRANSFAC database on potential transcription factor recognition sites (BIOBASE Corporation, Wolfenbuettel, Germany) as well as by using the rSNPBase database of curated regulatory SNPs (http://rsnp.psych.ac.cn) [35].

## 3. Results

### 3.1. Association Study between the *CYP4A11* and *CYP4F2* SNPs and CAD Risk

The genotype and allele frequencies of *CYP4A11* and *CYP4F2* SNPs are shown in Table 2. A significant departure from Hardy–Weinberg equilibrium (HWE) was observed for SNP rs9333029 of *CYP4A11*: no individuals with homozygous genotype GG were identified among the study participants. Notably, frequencies of genotypes AA and AG were compatible with those reported in various European populations, and genotype GG is also uncommon among Europeans (the 1000 Genomes Project, http://www.internationalgenome.org). Allele and genotype frequencies of other SNPs were similar with those observed in other European populations. As can be seen from Table 2, polymorphism rs9332978 of *CYP4A11* was found to be associated with increased risk of coronary artery disease at codominant genetic model after adjustment for confounding factors. In particular, the increased risk of CAD was associated with a carriage of variant allele C (*P* = 0.002, *Q* = 0.01) and genotypes T/C and CC (*P* = 0.008, *Q* = 0.04). In addition, allele G of rs3890011 showed a significant association with the risk of CAD (*P* = 0.02, *Q* = 0.05). These associations remain significant after adjustment for multiple testing using the FDR method.  Table 3 shows gender-stratified distributions of genotypes and alleles for the studied SNPs in the case and control groups. The rs3890011 and rs9332978 polymorphisms of *CYP4A11* were associated with the increased risk of coronary artery disease exclusively in females (*P* = 0.004, *Q* = 0.01).

### 3.2. Interactions between SNPs of *CYP4A11* and *CYP4F2*

We performed a log-likelihood ratio test to look for epistatic interaction between SNPs (Table 4). As can be seen from Table 4, SNPs rs3890011 (*P* = 0.035) and rs9332978 (*P* = 0.004) of *CYP4A11* showed significant individual effects on CAD risk at a dominant genetic model. Notably, we found epistatic interactions between rs9332978 of *CYP4A11* and rs1558139 of *CYP4F2* (recessive model, *P*_interaction_ = 0.025) as well as between rs3093098 and rs1558139 of the *CYP4F2* gene (overdominant model, *P*_interaction_ = 0.047).

### 3.3. Analysis of Haplotypes and Linkage Disequilibrium between SNPs

The three SNPs of the *CYP4A11* gene and the two SNPs of *CYP4F2* were used to establish five haplotypes. The patterns of estimated haplotypes and their frequencies in the case and control groups are shown in Table 5. Four haplotypes of *CYP4A11* gene and three haplotypes of *CYP4F2* with a frequency > 1% have been identified in the study patients. As can be seen from Table 5, the overall distribution of the haplotypes of *CYP4A11* was significantly different between the CAD patients and the healthy controls (*P* = 0.019). The frequency of the H2 (G-C-A) haplotype was significantly higher in the CAD patients than that in the healthy controls (OR = 1.42, 95% CI: 1.13–1.79, *P* = 0.003). Thus, the common G-C-A haplotype was thought to be a susceptibility haplotype in CAD patients. No significant difference in the *CYP4F2* haplotype frequencies was found between the case and control groups (*P* > 0.05).  Table 6 shows pairwise linkage disequilibrium coefficients among the SNPs of *CYP4A11*. Polymorphism rs9332978 in the promoter of *CYP4A11* was in strong linkage disequilibrium to the intronic polymorphism rs3890011 (*D*′ = 0.974, *P* < 0.0001). A strong linkage disequilibrium was also found between SNPs rs3890011 and rs9333029 of *CYP4A11* (*D*′ = 0.955, *P* < 0.0001). Furthermore SNPs rs9332978 and rs9333029 are also in linkage disequilibrium but with a lesser degree (*D*′ = 0.565, *P* < 0.0001).

### 3.4. In Silico Analysis of SNPs

Table 6 shows the results of bioinformatic analysis for the regulatory potential of the studied SNPs. The SNP Function Prediction tool allowed identifying putative transcription factor binding sites (TFBS) at SNP rs9332978 of *CYP4A11* and SNP rs3093098 of *CYP4F2.* In particular, 27 and 7 TFBSs were identified to possess the potential impact on the gene expression through a binding site located at SNP rs9332978 in the proximal promoter of *CYP4A11*, as reported by the TRANSFAC database and rSNPBase, respectively. As can be seen from Table 6 (detailed information on all transcription factor binding sites identified is listed in Supplementary Tables 1 and 2), polymorphism rs3890011 of *CYP4A11* has the regulatory potential and an experimentally proven eQTL (i.e., locus controlling transcript levels of the gene). Moreover, SNPs rs3093098 and rs1558139 of *CYP4F2* fall into RNA binding protein-mediated regulation sites.

## 4. Discussion

### 4.1. Variation in the *CYP4A11* Gene and CAD Susceptibility

*CYP4A11* and *CYP4F2* are highly polymorphic genes which became attractive candidates for association studies of cardiovascular diseases. A number of these studies have been done in Asian populations, and no studies investigated the contribution of the genes to coronary artery disease susceptibility in Europeans. In addition, the results of these studies were variable and sometimes contradictory, thus justifying the need for further investigations of the relationship between *CYP4A11* and *CYP4F2* gene polymorphisms and CAD risk in independent racial and ethnic groups.

SNP T8590C (rs1126742) is the most extensively investigated polymorphism in the *CYP4A11* gene that has been shown to be associated with the level of blood pressure and hypertension susceptibility [17, 36] as well as with endothelial dysfunction in the coronary arteries in patients with CAD in Europeans [37]. Another SNP rs3890011 in intron of *CYP4A11* was also a subject of investigations in cardiovascular disorders in various populations of the world. In particular, a study in Chinese population did not identify the link of the rs3890011 polymorphism with blood pressure variation and hypertension susceptibility [38]. However, Fu with coworkers have revealed a significant association between SNP rs3890011 and the risk of CAD, but the association occurred only in males [24]. This study did not observe an association between a promoter polymorphism rs9332978 of *CYP4A11* and CAD risk. The authors also reported that the functional effect of the rs3890011 polymorphism is related to neighboring functional SNPs (possibly rs9332978), potentially affecting the structure and/or catalytic activity of the enzyme [24].

The present study was designed to investigate whether common SNPs rs3890011, rs1126742, rs9332978, and rs9333029 of *CYP4A11* and rs3093098 and rs1558139 *CYP4F2* are associated with the risk of CAD in Russian population. The present study has revealed that polymorphisms rs3890011 and rs9332978 are both associated with the risk of CAD; however, the association occurred only in females. Interestingly, these SNPs represent a part of common functional haplotype of *CYP4A11*, as it has been demonstrated by our and some other studies [23, 39]. In particular, haplotype G-G-T (rs9332978, rs3890011, rs1126742) was found to be moderately associated with the CAD risk in Chinese Han population, whereas SNP rs9332978 alone did not show a significant association with disease risk [39]. We found that the high CAD risk haplotype G-C-A (rs3890011, rs9332978, and rs9333029; risk alleles are underlined) in our patients coincides with a part of haplotype G-C-T (rs3890011, rs9332978, rs1126742) reported as the disease risk haplotype in the study of Fu with coworkers [39]. It seems reasonable to say that the functional effect of this haplotype on enzyme's activity could be related with variation in the proximal promoter of *CYP4A11*, that is, with SNP rs9332978. At least, this suggestion may be supported by the study in Japanese population that reported a relationship between the SNP rs9332978, expression of *CYP4A11*, and the hypertension risk [40]. Sugimoto et al. [40] observed that the −845GG genotype is associated with lower promoter activity when compared with −845AA genotype, and allele −845G was positively correlated to hypertension susceptibility. Additionally, the authors [40] supposed that the rs9332978 polymorphism falls into DNA binding site for an unidentified protein and/or potential transcription factor. The bioinformatic analysis for the regulatory potential of investigated polymorphisms allowed us identifying putative transcription factor binding sites at SNP rs9332978 of the *CYP4A11* gene. Interestingly, it was found that the DNA binding site located at this SNP may be regulated by numerous transcription factors representing the pathways being involved into the molecular mechanisms of coronary atherosclerosis. In particular, CEBPB is an important transcription factor binding to promoter regions of multiple inflammatory response genes, synergistically upregulating and sustaining their expression after inflammatory stimulation [41, 42]. HNF4A (hepatocyte nuclear factor 4 alpha) is a transcriptionally controlled transcription factor that binds to DNA sites required for the transcription of alpha 1-antitrypsin, apolipoprotein CIII, transthyretin genes, and HNF1-alpha (data obtained from the UniProtKB). In accordance with Gene Ontology descriptions (GO, http://www.geneontology.org), HNF4A has the potential to coregulate genes involved in blood coagulation, lipid homeostasis (positive regulation of cholesterol homeostasis), cell proliferation, and other important biological processes which play a role in the pathogenesis of CAD. Altogether, these data make SNP rs9332978 of *CYP4A11* a subject of great interest for further research of molecular pathogenesis of coronary artery disease.

### 4.2. Possible Role of SNP rs9332978 of *CYP4A11* in the Pathogenesis of CAD

**Figure 1:**
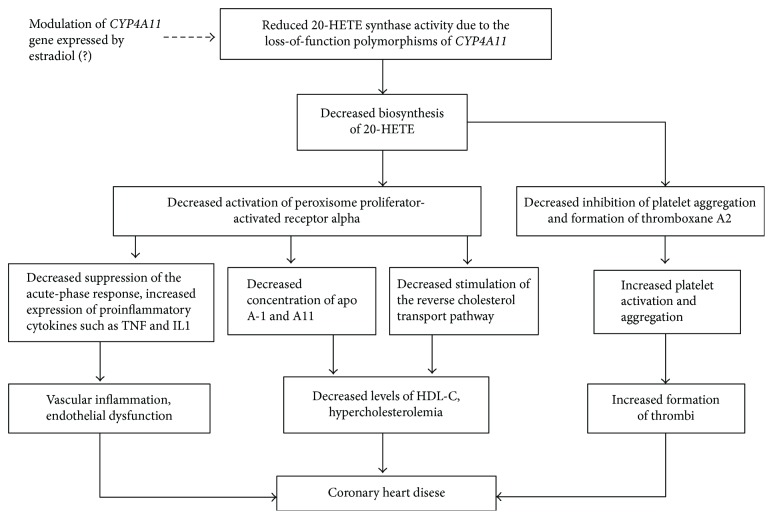
Proposed mechanisms by which the loss-of-function polymorphisms of the *CYP4A11* gene are involved in the pathogenesis of coronary heart disease (see the text for details).

Literature data on a relationship between decreased 20-HETEs and pathogenesis of cardiovascular disease are extremely limited. Nevertheless, based on available publications, the UniProtKB database and Gene Ontology descriptions, we proposed the mechanisms by which loss-of-function polymorphisms rs9332978 and rs1126742 of *CYP4A11* are associated with reduced 20-HETE synthase activity and increased risk of coronary artery disease (Figure 1). 20-HETE is a potent endogenous agonist of PPAR*α* (peroxisome proliferator-activated receptor alpha) [43]. The peroxisome proliferator-activated receptors are ligand-activated transcription factors belonging to the nuclear hormone receptor superfamily. Bioinformatic analysis allowed us identifying a binding site at SNP rs9332978 for transcription factor PPARG as a potent coregulator of *CYP4A11* gene expression (Table 6). Importantly, PPAR*α* is a major regulator of intra- and extracellular lipid metabolism [44]. PPAR*α* and PPARG serve as physiological sensors of lipid levels whereby dietary fatty acids can modulate lipid homeostasis [45]. PPAR*α* activation can increase the levels of HDL-C through increasing concentration of apo A-I and A-II and through stimulating the reverse cholesterol transport pathway [46]. Hence, a deficiency of 20-HETE may reduce the hypolipidemic effects of PPAR*α*, leading to decreased HDL-C and hypercholesterolemia. Moreover, activation of PPAR*α* can exert anti-inflammatory effects, suppressing the acute-phase response and decreasing the release of proinflammatory cytokines [47]. In addition, 20-HETE was found to be a potent, dose-dependent inhibitor of platelet aggregation and biosynthesis of thromboxane A2, most probably by antagonizing the prostaglandin H2/thromboxane A2 (PGH2/TXA2) receptor [48], thereby leading to increased formation of thrombi.

However, when interpreting genotype-phenotype correlation, it is important to keep in mind that it is difficult to predict the consequences of a change in the activity of the CYP4A11 enzyme, especially taking the dual role of 20-HETE in vascular and renal homeostasis into consideration [12, 15, 49]. Undoubtedly, this means that our suggestions on the involvement of these SNPs in CAD pathogenesis require experimental confirmation. In addition, we cannot rule out the possibility of complex interactions between different polymorphic genes and their comprehensive contribution to the levels of 20-HETE in the heart and coronary arteries in patients with CAD. In this context, our interesting finding was an epistatic interaction between SNP rs9332978 of *CYP4A11* and SNP rs1558139 of *CYP4F2* (this polymorphism was associated with the risk of essential hypertension in Japanese population [19]) in CAD patients suggesting that gene-gene interactions could be involved into the regulation of 20-HETE metabolism and jointly contribute to the development of coronary artery disease.

### 4.3. Gender-Specific Relationship between SNP rs9332978 of *CYP4A11* and Risk of CAD

Interestingly, the effects of rs9332978 of the *CYP4A11* gene on CAD risk in our study were evident only in women. This finding supports the hypothesis that the interaction of sex hormones with expression cytochrome P450 enzymes involved in the 20-HETE metabolism could have a role in well-established sex dimorphism in the risk of cardiovascular disease [50]. It can be assumed that association between rs9332978 of *CYP4A11* and CAD susceptibility in women may be related with the inhibitory effect of estradiol on the *CYP4A11* expression in the carriers of the variant genotypes, leading to reduced synthesis of 20-HETE and increasing the disease risk through the mechanisms described above. Biosynthesis of 20-HETE is regulated in age- and sex-dependent manner [15, 49, 51], and CYP4A11 itself has a catalytic activity for the metabolism of estrogens such as 17beta-estradiol and estrone [52, 53]. This means that estrogens may represent important modifiers of CYP4A11-medicated metabolism of 20-HETE. The study of White with coworkers provided evidence that polymorphism in the *CYP4A11* gene is related with disorders underlying coronary atherosclerosis, and this relationship is also sex specific [54]. In particular, polymorphism rs1126742 of *CYP4A11* was found to be associated with HDL-C and C-reactive protein in women [54]. This relationship may be explained by the effects of PPAR*α* agonists which are known to improve lipid metabolism disorder, and this capacity appears to be modulated by estrogens [55]. White with coworkers proposed that the effect of the loss-of-function allele of *CYP4A11* on decreased formation of PPAR*α* agonists could be magnified in women [54]. SNP rs9332978 of *CYP4A11* is located within a binding site for transcription factor FOXA1 (forkhead box A1 or hepatocyte nuclear factor 3 alpha). Interestingly, FOXA1 modulates the transcriptional activity of nuclear hormone receptors and is involved in positive regulation of intracellular estrogen receptor signaling pathway [56]. Apparently, female hormones have the potential to bind with specific sites at the promoter of *CYP4A11* and therefore could be responsible for sex-specific alterations in the expression of CYP4A11, thereby affecting the production of 20-HETE. The mechanisms whereby estrogens exert their regulatory effects on CAD through the modulation of *CYP4A11* gene expression remained to be elucidated in further studies.

There are several limitations to address in the context of the current results. First, we did not measure 20-HETE levels in the study patients, and possible alterations in the metabolism of arachidonic acid in CAD could not be established in our study. Further studies are needed to clarify the effects of the investigated polymorphisms of *CYP4A11* on arachidonic acid metabolism and/or 20-HETE production. Second, other polymorphisms in the *CYP4A11* gene such as those located in intronic regions or distal promoter regions not investigated in this study might also be associated with disease susceptibility. Unfortunately, insufficient genotyping call rate (<70%) for polymorphism rs1126742 (T8590C) of *CYP4A11* did not allow the inclusion of this SNP into the study. In order to describe the complete haplotype structure of the *CYP4A11* gene, it is necessary to expend a spectrum of polymorphisms in future studies. Third, potential interactions of genetic polymorphisms of *CYP4A11* and *CYP4F2* with environmental conditions such as food and dietary elements were unexplored in the study, thus not allowing any conclusion to be drawn with respect to sex-specific associations between the genes and disease risk.

## 5. Conclusions

The present study identified that polymorphism rs9332978 of *CYP4A11* could be a novel marker of genetic susceptibility to coronary artery disease, at least in Europeans. Moreover, our study provided additional evidence that *CYP4A11* is an important susceptibility gene for coronary artery disease despite the fact that different polymorphisms of the gene showed association with disease risk in various populations. Although the molecular mechanisms underlying the development of coronary artery disease in women with the rs9332978 polymorphism of *CYP4A11* remain to be determined, the results of the present study support the hypothesis that variation in the *CYP4A11* gene is an important determinant associated with the risk of coronary artery disease in gender-specific manner. Further efforts should be made to address the function of the studied SNPs of *CYP4A11* in arachidonic acid metabolism in order to determine the effect of the polymorphisms on the production of 20-HETE in the coronary arteries in CAD patients. Nevertheless, the association between SNP rs9332978 of *CYP4A11* and the risk of coronary artery disease provides insights into the molecular basis of disease pathogenesis and suggests possible avenues in developing novel drugs for pharmacological intervention in the metabolism of 20-hydroxyeicosatetraenoic acids in patients with CVD. Further pharmacogenomic studies are needed to substantiate the contribution of CYP4A11 polymorphisms in the pathogenesis of coronary artery disease and to assess the dual role of 20-hydroxyeicosatetraenoic acids in cardiovascular homeostasis as a promising target for vascular medicine in the future.

## Figures and Tables

**Table 1:** Demographic and clinical data of the study participants.

Baseline characteristics	Controls, *n* = 686	CAD patients, *n* = 637	*P* value
Age, mean ± SD	58.8 ± 7.6	59.5 ± 10.3	0.16
Males	404 (58.9)	401 (63.0)	0.13
BMI (kg/m^2^), mean ± SD	27.9 ± 10.2	28.8 ± 9.4	0.10
Hypertension	0 (0.0)	842 (90.2)	**—**
Diabetes	0 (0.0)	79 (8.5)	**—**
Fasting blood glucose (mmol/L)^1^	5.2 ± 0.7	8.1 ± 0.5	**<0.0001**
TC (mmol/L)^1^	4.2 ± 0.3	6.2 ± 0.4	**<0.0001**
HDL-C (mmol/L)^1^	1.2 ± 0.2	1.1 ± 0.3	**<0.0001**
LDL-C (mmol/L)^1^	3.1 ± 0.3	4.3 ± 0.6	**<0.0001**
TG (mmol/L)^1^	1.5 ± 0.4	1.7 ± 0.3	**<0.0001**
Smokers (ever/never)^2^	273 (41.5)	251 (43.5)	0.48
Positive family history of CAD	150 (23.8)	323 (34.6)	**<0.0001**
Positive family history of hypertension	100 (15.9)	273 (29.2)	**<0.0001**
Positive family history of diabetes	24 (3.8)	127 (13.6)	**<0.0001**

^1^The biochemical parameters were available from 347 subjects of the control group. ^2^Data on smoking status were not available from 60 CAD patients and 28 controls. SD: standard deviation; BMI: body mass index (age and BMI were normally distributed and were analyzed by Student's *t*-test); TC: total cholesterol; HDL-C: high-density lipoprotein cholesterol; LDL-C: low-density lipoprotein cholesterol; TG: triglyceride. Other data are expressed as frequencies and percentages and were evaluated by the *χ* ^2^ test. Bolded is statistically significant *P* value.

**Table 2:** Genotype and allele frequencies for SNPs of *CYP4A11* and *CYP4F2* in patients with CAD and healthy controls.

Gene, polymorphism	Genotype, allele	Controls, *n* = 686 *n* (%)^1^	CAD patients, *n* = 637 *n* (%)^1^	OR (95% CI)^2^	*P* value	*Q* value
*CYP4A11*, C>G (rs3890011)	C/C	404 (58.9)	338 (53.1)	1.00	0.086	0.22
	C/G	236 (34.4)	244 (38.3)	1.21 (0.91–1.58)		
	G/G	46 (6.7)	55 (8.6)	1.40 (0.92–2.23)		
	G	328 (23.9)	354 (27.8)	1.21 (1.02–1.48)	0.02	0.05
*CYP4A11*, T>C (rs9332978)	T/T	542 (79)	459 (72.3)	1.00	0.008	0.04
	T/C	134 (19.5)	158 (24.9)	1.42 (1.09–1.84)		
	C/C	10 (1.5)	18 (2.8)	2.26 (1.04–4.95)		
	C	154 (11.2)	194 (15.3)	1.44 (1.16–1.81)	0.002	0.01
*CYP4A11*, A>G (rs9333029)	A/A	526 (76.9)	477 (74.9)	1.00	0.43	0.72
	A/G	158 (23.1)	160 (25.1)	1.09 (0.78–1.59)		
	G/G	0 (0.0)	0 (0.0)	—		
	G	158 (11.5)	160 (12.6)	1.04 (0.80–1.43)	0.42	0.70
*CYP4F2*, A>G (rs3093098)	A/A	479 (69.8)	448 (70.3)	1.00	0.89	0.91
	A/G	186 (27.1)	172 (27)	0.97 (0.76–1.27)		
	G/G	21 (3.1)	17 (2.7)	0.85 (0.43–1.68)		
	G	228 (16.6)	206 (16.2)	0.96 (0.80–1.17)	0.76	0.76
*CYP4F2*, G>A (rs1558139)	G/G	200 (29.1)	192 (30.1)	1.00	0.91	0.91
	G/A	336 (49)	312 (49)	0.98 (0.73–1.28)		
	A/A	150 (21.9)	133 (20.9)	0.94 (0.70–1.29)		
	A	636 (46.4)	578 (45.4)	0.97 (0.80–1.18)	0.61	0.76

^1^Absolute number and percentage of individuals/chromosomes with particular genotype/allele. ^2^Odds ratio with 95% confidence intervals adjusted for age, gender, BMI, hypertension, diabetes, and smoking.

**Table 3:** Genotype frequencies for SNPs of the *CYP4A11* and *CYP4F2* genes in patients with CAD and healthy controls stratified by gender.

Gene, polymorphism	Genotype	Males, *n* (%)^1^					Females, *n* (%)^1^				
		Controls, *n* = 404	CAD patients, *n* = 401	*P* value	*Q* value	_adj_OR (95% CI)^2^	Controls, *n* = 282	CAD patients, *n* = 236	*P* value	*Q* value	_adj_OR (95% CI)^2^
*CYP4A11*, C>G (rs3890011)	C/C	228 (56.4)	224 (55.9)	0.98	0.98	1.00	176 (62.4)	114 (48.3)	0.004	0.01	1.00
	C/G	144 (35.6)	144 (35.9)			1.01 (0.76–1.37)	92 (32.6)	100 (42.4)			1.62 (1.14–2.39)
	G/G	32 (7.9)	33 (8.2)			1.04 (0.61–1.77)	14 (5.0)	22 (9.3)			2.66 (1.29–5.46)
*CYP4A11*, T>C (rs9332978)	T/T	313 (77.5)	297 (74.1)	0.45	0.98	1.00	229 (81.2)	162 (69.2)	0.004	0.01	1.00
	T/C	86 (21.3)	96 (23.9)			1.18 (0.85–1.62)	48 (17.0)	62 (26.5)			1.85 (1.21–2.82)
	C/C	5 (1.2)	8 (2.0)			1.69 (0.52–5.24)	5 (1.8)	10 (4.3)			3.09 (1.02–9.35)
*CYP4A11*, A>G (rs9333029)	A/A	300 (74.4)	302 (75.3)	0.77	0.98	1.00	226 (80.4)	175 (74.2)	0.12	0.20	1.00
	A/G	103 (25.6)	99 (24.7)			0.98 (0.62–1.47)	55 (19.6)	61 (25.8)			1.44 (0.89–2.76)
	G/G	0 (0.0)	0 (0.0)			—	0 (0.0)	0 (0.0)			—
*CYP4F2*, A>G (rs3093098)	A/A	277 (68.6)	278 (69.3)	0.33	0.98	1.00	202 (71.6)	170 (72.0)	0.54	0.54	1.00
	A/G	112 (27.7)	115 (28.7)			1.02 (0.74–1.39)	74 (26.2)	57 (24.2)			0.93 (0.61–1.84)
	G/G	15 (3.7)	8 (2.0)			0.55 (0.24–1.35)	6 (2.1)	9 (3.8)			1.72 (0.61–5.97)
*CYP4F2*, G>A (rs1558139)	G/G	124 (30.7)	124 (30.9)	0.95	0.98	1.00	76 (27.0)	68 (28.8)	0.46	0.54	1.00
	G/A	194 (48.0)	188 (46.9)			0.96 (0.72–1.38)	142 (50.4)	124 (52.5)			1.02 (0.64–1.85)
	A/A	86 (21.3)	89 (22.2)			1.04 (0.72–1.56)	64 (22.7)	44 (18.6)			0.77 (0.44–1.34)

^1^Absolute number and percentage of individuals with particular genotype. ^2^Odds ratio with 95% confidence intervals adjusted for age, BMI, hypertension, diabetes, and smoking.

**Table 4:** Epistatic interactions between the *CYP4A11* and *CYP4F2* genes in CAD (gene-gene interactions are evaluated by SNPassoc package for R [31]).

SNPs	Genetic models	*CYP4A11* (rs3890011)	*CYP4A11* (rs9332978)	*CYP4A11* (rs9333029)	*CYP4F2* (rs3093098)	*CYP4F2* (rs1558139)
*CYP4A11* (rs3890011)	Сodominant	0.086	0.459	0.725	0.598	0.435
	Dominant	**0.035**	0.712	0.450	0.319	0.533
	Recessive	0.180	0.159	—	0.986	0.594
	Overdominant	0.152	0.879	0.168	0.899	0.844

*CYP4A11* (rs9332978)	Сodominant	0.933	**0.008**	0.396	0.194	0.226
	Dominant	0.768	**0.004** ^∗^	0.885	0.156	0.512
	Recessive	0.608	0.063	—	—	**0.025**
	Overdominant	0.824	**0.019**	0.935	0.307	0.686

*CYP4A11* (rs9333029)	Сodominant	0.477	0.371	0.433	0.902	0.216
	Dominant	0.534	0.398	0.433	0.835	0.098
	Recessive	—	—	—	—	—
	Overdominant	0.899	0.441	0.433	0.743	0.134

*CYP4F2* (rs3093098)	Сodominant	0.849	0.837	0.866	0.894	0.096
	Dominant	0.627	0.592	0.711	0.775	0.152
	Recessive	0.675	0.703	—	0.661	—
	Overdominant	0.808	0.718	0.838	0.897	**0.047**

*CYP4F2* (rs1558139)	Сodominant	0.890	0.923	0.918	0.806	0.912
	Dominant	0.715	0.807	0.718	0.668	0.733
	Recessive	0.701	0.764	—	0.686	0.717
	Overdominant	0.998	0.913	0.940	0.991	0.989

The upper part of the matrix contains the *P* values for epistatic interactions evaluated by log-likelihood ratio (LRT) test. The diagonal contains the *P* values from LRT for the crude effect of each SNP. The lower triangle contains the *P* values from LRT comparing the two-SNP additive likelihood to the best of the single-SNP models. Bolded are statistically significant *P* values for SNP-SNP interactions (∗most significant *P* value for a particular model). *P* values are adjusted for age and gender.

**Table 5:** Estimated haplotype frequencies of *CYP4A11* in CAD patients and controls.

Haplotypes^1^		Controls	CAD patients	OR (95% CI)^2^	*P* value
SNPs C>G (rs3890011), T>C (rs9332978), and A>G (rs9333029) of *CYP4A11*					
H1	C-T-A	0.7549	0.7158	1.00	—
H2	G-C-A	0.1060	0.1433	1.41 (1.12–1.78)	**0.0036**
H3	G-T-G	0.1087	0.1146	1.15 (0.88–1.50)	0.30
H4	G-T-A	0.0207	0.0135	0.67 (0.37–1.22)	0.19
Global haplotype association *P* value: **0.021**					

SNPs A>G (rs3093098) and G>A (rs1558139) of *CYP4F2*					
H1	A-A	0.4636	0.4525	1.00	—
H2	A-G	0.3703	0.3858	1.05 (0.91–1.27)	0.49
H3	G-G	0.1662	0.1605	0.97 (0.78–1.24)	0.85
Global haplotype association *P* value: 0.72					

^1^Rare haplotypes with frequency < 0.01 are not shown. ^2^Odds ratio with 95% confidence intervals adjusted for age, gender, BMI, diabetes, and hypertension. Bolded is statistically significant *P* value.

**Table 6:** Bioinformatic analysis for the regulatory potential of the studied SNPs.

SNP	Allele	Location	SNP Function Prediction (FuncPred)^1^			Regulatory annotations on SNPs (rSNPBase)^2^							Transcription factors potentially related with SNP	
			TFBS	miRNA	Regulatory potential	rSNP	LD-proxy of rSNP (*r* ^2^ > 0.8)	Proximal regulation	Distal regulation	miRNA regulation	RNA binding protein- mediated regulation	eQTL	TRANSFAC database	rSNPBase
rs3890011	C/G	Intron	No	No	Yes	No	Yes	No	No	No	No	Yes	—	—
rs9332978	T/C	Promoter	Yes	No	No	Yes	Yes	Yes	No	No	No	No	AIRE, ATF6, CDPCR3, CEBPA, CEBPDELTA, CEBPGAMMA, CEBP, CRX, FAC1, GRE, IPF1, MYOGNF1, OCT1, OCT4, OCT, PAX3, PAX6, PAX8, PLZF, POU3F2, PPARG, S8, SP3, SREBP1, SREBP, TAXCREB, ZF5	FOXA1, HNF4A, ARID3A, CEBPB
rs9333029	A/G	Intron	No	No	No	No	Yes	No	No	No	No	Yes	—	—
rs3093098	A/G	Intron	Yes	No	No	Yes	Yes	Yes	Yes	No	Yes	No	AP4, CACD, DR4, ETF, GABP, GRE, HAND1E47, HIC1, MYOGNF1, PAX3, PLZF, PPARA, RFX, SP1, SP3, SPZ1, SZF11, TAXCREB, TBX5, TEL2, VDR, WT1, ZF5	Pol2
rs1558139	G/A	Intron	No	No	Yes	Yes	Yes	Yes	No	No	Yes	Yes	—	—

^1^Data predicted by the SNP Function Prediction tool, National Institute of Environmental Health Sciences (https://snpinfo.niehs.nih.gov/snpinfo/). TFBS: transcription factor binding site; ND: no data. ^2^Data obtained at rSNPBase, a database of curated regulatory SNPs (http://rsnp.psych.ac.cn). rSNP, rSNPBase identified regulatory SNPs; LD-proxy of rSNP (*r* ^2^ > 0.8), SNP in strong LD with rSNPs; proximal regulation, SNP involved in proximal transcriptional regulation; distal regulation, SNP involved in distal transcriptional regulation; miRNA regulation, SNP within mature miRNA; RNA binding protein mediated regulation, SNP involved in RNA binding protein-mediated post-transcriptional regulation; eQTL, SNP with experimental eQTL evidence. TRANSFAC is the database on potential transcription factor recognition sites (BIOBASE Corporation, Wolfenbuettel, Germany).

## Supplementary Materials

Supplementary Materials Supplementary Table 1: Transcription Factor Binding Sites for SNP rs9332978 of *CYP4A11*. Supplementary Table 2: Transcription Factor Binding Sites for SNP rs9332978 of *CYP4A11*.
