# Supplementary material for: A Novel Polymorphism in the Promoter of the CYP4A11 Gene Is Associated with Susceptibility to Coronary Artery Disease
Source: Dis Markers. 2018 Feb 1;2018:5812802. doi: 10.1155/2018/5812802 (PMC5816861; doi:10.1155/2018/5812802)
Supplement: Supplementary Materials — Supplementary Table 1: Transcription Factor Binding Sites for SNP rs9332978 of CYP4A11. Supplementary Table 2: Transcription Factor Binding Sites for SNP rs9332978 of CYP4A11. [file 5812802.f1.doc]

**Supplementary Information**: This file contains the following SI:

Supplementary table 1 Transcription Factor Binding Sites for SNP rs9332978 of *CYP4A11* Supplementary table 2 Transcription Factor Binding Sites for SNP rs9332978 of *CYP4A11*

**Supplementary table 1 Transcription Factor Binding Sites for SNP rs9332978 of *CYP4A11* (TRANSFAC database)**

| SNP | Allele | Position | Prediction Strand | Forward Sequence | Core Match Score | Matrix Match Score | [Transfac Matrix ID](http://www.gene-regulation.com/) |
| --- | --- | --- | --- | --- | --- | --- | --- |
| rs9332978 | C | 16 | - | caccctacaatagtacgtAATTAca | 0.772 | 0.542 | V$AIRE_02 |
| rs9332978 | C | 4 | - | gtaCGTAA | 0.8 | 0.677 | V$ATF6_01 |
| rs9332978 | T | 13 | + | cctacaatagTATGT | 0.744 | 0.613 | V$CDPCR3_01 |
| rs9332978 | T | 9 | - | CAATAgtatgtaatt | 0.995 | 0.643 | V$CDPCR3_01 |
| rs9332978 | T | 14 | - | CCCTAcaatagtatg | 0.744 | 0.597 | V$CDPCR3_01 |
| rs9332978 | C | 9 | - | CAATAgtacgtaatt | 0.995 | 0.647 | V$CDPCR3_01 |
| rs9332978 | C | 14 | - | CCCTAcaatagtacg | 0.744 | 0.521 | V$CDPCR3_01 |
| rs9332978 | T | 7 | - | atagtatGTAATta | 0.966 | 0.896 | V$CEBPA_01 |
| rs9332978 | C | 7 | - | atagtacGTAATta | 0.966 | 0.896 | V$CEBPA_01 |
| rs9332978 | T | 6 | - | tagtatGTAATt | 0.976 | 0.882 | V$CEBPDELTA_Q6 |
| rs9332978 | C | 6 | - | tagtacGTAATt | 0.976 | 0.885 | V$CEBPDELTA_Q6 |
| rs9332978 | T | 5 | - | agtatGTAATtac | 0.907 | 0.827 | V$CEBPGAMMA_Q6 |
| rs9332978 | C | 5 | - | agtacGTAATtac | 0.907 | 0.716 | V$CEBPGAMMA_Q6 |
| rs9332978 | C | 8 | - | aatagTACGTaat | 0.721 | 0.658 | V$CEBPGAMMA_Q6 |
| rs9332978 | T | 7 | + | atagtaTGTAAt | 0.990 | 0.949 | V$CEBP_Q3 |
| rs9332978 | C | 7 | + | atagtaCGTAAt | 0.979 | 0.939 | V$CEBP_Q3 |
| rs9332978 | C | 4 | - | gtaCGTAAttac | 0.832 | 0.792 | V$CREB_02 |
| rs9332978 | T | 3 | + | tatgTAATTacac | 0.955 | 0.923 | V$CRX_Q4 |
| rs9332978 | T | 2 | - | atgtAATTAcaca | 0.955 | 0.921 | V$CRX_Q4 |
| rs9332978 | C | 3 | + | tacgTAATTacac | 0.955 | 0.924 | V$CRX_Q4 |
| rs9332978 | C | 2 | - | acgtAATTAcaca | 0.955 | 0.899 | V$CRX_Q4 |
| rs9332978 | T | 14 | + | ccctACAATagtat | 0.906 | 0.790 | V$FAC1_01 |
| rs9332978 | C | 14 | + | ccctACAATagtac | 0.906 | 0.783 | V$FAC1_01 |
| rs9332978 | T | 10 | + | acaatagtatGTAATt | 0.713 | 0.657 | V$GRE_C |
| rs9332978 | C | 6 | - | tAGTACgtaattacac | 0.951 | 0.666 | V$GRE_C |
| rs9332978 | T | 3 | + | tatgTAATTaca | 0.868 | 0.818 | V$IPF1_Q4 |
| rs9332978 | T | 1 | - | tgtAATTAcaca | 0.868 | 0.802 | V$IPF1_Q4 |
| rs9332978 | C | 3 | + | tacgTAATTaca | 0.868 | 0.817 | V$IPF1_Q4 |
| rs9332978 | C | 1 | - | cgtAATTAcaca | 0.868 | 0.786 | V$IPF1_Q4 |
| rs9332978 | T | 26 | - | aattctctttcaCCCTAcaatagtatgta | 0.688 | 0.508 | V$MYOGNF1_01 |
| rs9332978 | C | 26 | - | aattctctttcaCCCTAcaatagtacgta | 0.688 | 0.501 | V$MYOGNF1_01 |
| rs9332978 | T | 8 | + | aatagTATGTaatta | 0.766 | 0.711 | V$OCT1_02 |
| rs9332978 | T | 13 | + | cctACAATagtat | 0.836 | 0.793 | V$OCT1_03 |
| rs9332978 | T | 3 | + | tatGTAATtacac | 1 | 0.988 | V$OCT1_03 |
| rs9332978 | T | 2 | - | atgtaATTACaca | 1 | 0.993 | V$OCT1_03 |
| rs9332978 | C | 13 | + | cctACAATagtac | 0.836 | 0.789 | V$OCT1_03 |
| rs9332978 | C | 3 | + | tacGTAATtacac | 1 | 0.990 | V$OCT1_03 |
| rs9332978 | C | 2 | - | acgtaATTACaca | 1 | 0.994 | V$OCT1_03 |
| rs9332978 | T | 3 | - | tatGTAATtac | 0.746 | 0.827 | V$OCT1_Q5_01 |
| rs9332978 | T | 9 | + | caatagtATGTAatt | 0.955 | 0.672 | V$OCT4_01 |
| rs9332978 | T | 3 | - | tATGTAattac | 0.963 | 0.845 | V$OCT_Q6 |
| rs9332978 | C | 9 | - | caatagtaCGTAAttacacaa | 0.868 | 0.663 | V$PAX3_B |
| rs9332978 | T | 12 | - | ctacaatagtaTGTAAttaca | 0.688 | 0.567 | V$PAX6_01 |
| rs9332978 | C | 12 | - | ctacaatagtaCGTAAttaca | 0.959 | 0.694 | V$PAX6_01 |
| rs9332978 | T | 11 | + | tacaatagTATGTaa | 0.736 | 0.722 | V$PAX8_01 |
| rs9332978 | T | 11 | + | tacaatagtatgTAATTacacaatcagaa | 0.735 | 0.555 | V$PLZF_02 |
| rs9332978 | T | 10 | - | acaatagtatgtAATTAcacaatcagaac | 0.735 | 0.621 | V$PLZF_02 |
| rs9332978 | T | 14 | - | ccctacaatagtATGTAattacacaatca | 0.735 | 0.578 | V$PLZF_02 |
| rs9332978 | C | 11 | + | tacaatagtacgTAATTacacaatcagaa | 0.735 | 0.543 | V$PLZF_02 |
| rs9332978 | C | 10 | - | acaatagtacgtAATTAcacaatcagaac | 0.735 | 0.624 | V$PLZF_02 |
| rs9332978 | T | 4 | + | GTATGtaatt | 0.868 | 0.707 | V$POU3F2_02 |
| rs9332978 | T | 8 | - | aatagTATGT | 0.456 | 0.597 | V$POU3F2_02 |
| rs9332978 | C | 4 | + | GTACGtaatt | 0.651 | 0.594 | V$POU3F2_02 |
| rs9332978 | T | 9 | - | caatagtatgtaaTTACAcaatc | 0.634 | 0.502 | V$PPARG_02 |
| rs9332978 | C | 9 | - | caatagtacgtaaTTACAcaatc | 0.634 | 0.522 | V$PPARG_02 |
| rs9332978 | T | 5 | + | agtatgtAATTAcaca | 1 | 0.959 | V$S8_01 |
| rs9332978 | T | 3 | - | tatgTAATTacacaat | 1 | 0.951 | V$S8_01 |
| rs9332978 | C | 5 | + | agtacgtAATTAcaca | 1 | 0.959 | V$S8_01 |
| rs9332978 | C | 3 | - | tacgTAATTacacaat | 1 | 0.955 | V$S8_01 |
| rs9332978 | T | 14 | - | cCCTACaatagtat | 0.943 | 0.840 | V$SP3_Q3 |
| rs9332978 | C | 5 | - | agtACGTAatt | 0.749 | 0.641 | V$SREBP1_01 |
| rs9332978 | T | 6 | - | taGTATGtaatt | 0.797 | 0.737 | V$SREBP_Q3 |
| rs9332978 | C | 5 | - | agtaCGTAAttacac | 0.905 | 0.709 | V$TAXCREB_01 |
| rs9332978 | C | 6 | - | taGTACGtaatta | 0.840 | 0.729 | V$ZF5_B |

Last columns shows putative transcription factor binding matrices as reported in accordance with the TRANSFAC database. Capital letters within the sequence indicate the position of the core string within the matching matrix.

**Supplementary table 2 Transcription Factor Binding Sites for SNP rs9332978 of *CYP4A11*** **(rSNPBase)**


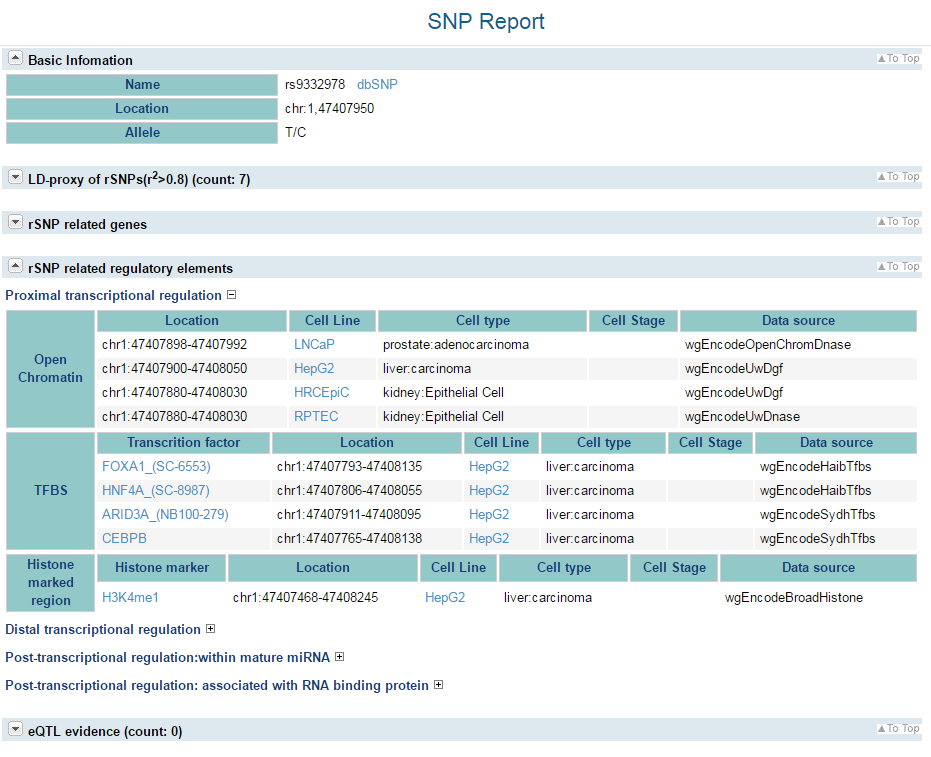


Data obtained at rSNPBase, a database of curated regulatory SNPs ([http://rsnp.psych.ac.cn](http://rsnp.psych.ac.cn/))
